# Supplementary material for: Establishment, Genetic Diversity, and Habitat Suitability of Aedes albopictus Populations from Ecuador
Source: Insects. 2022 Mar 19;13(3):305. doi: 10.3390/insects13030305 (PMC8950245; doi:10.3390/insects13030305)
Supplement: Supplementary file 1 [file insects-13-00305-s001.zip › insects-1614802-supplementary.pdf]

Table S1. Haplotype designation for individuals of *Aedes albopictus* collected in six localities of Ecuador

Accession N°	ECUs	Organism	Country	Locality	Latitude	Longitude	Collection Date	Sex	Haplotype	Trap	Reference
OK266899	ECU27416	*Aedes albopictus*	Ecuador	Cachaco	0,8337	-78,4023	24/10/2018	Female	H2	Ovitrap	This study
OK266900	ECU27417	*Aedes albopictus*	Ecuador	Cachaco	0,8337	-78,4023	24/10/2018	Male	H2	Ovitrap	This study
OK266901	ECU27418	*Aedes albopictus*	Ecuador	Cachaco	0,8337	-78,4023	24/10/2018	Male	H2	Ovitrap	This study
OK266902	ECU27419	*Aedes albopictus*	Ecuador	Cachaco	0,8337	-78,4023	12/05/2018	Male	H2	Ovitrap	This study
OK266903	ECU27420	*Aedes albopictus*	Ecuador	Cachaco	0,8337	-78,4023	12/05/2018	Male	H2	Ovitrap	This study
OK266904	ECU27421	*Aedes albopictus*	Ecuador	Cachaco	0,8337	-78,4023	13/02/2019	Female	H2	Ovitrap	This study
OK266905	ECU27422	*Aedes albopictus*	Ecuador	Cachaco	0,8337	-78,4023	13/02/2019	Female	H2	Ovitrap	This study
OK266906	ECU27423	*Aedes albopictus*	Ecuador	Cachaco	0,8337	-78,4023	13/02/2019	Male	H2	Ovitrap	This study
OK266907	ECU27424	*Aedes albopictus*	Ecuador	Cachaco	0,8337	-78,4023	07/11/2018	Female	H2	Ovitrap	This study
OK266908	ECU27425	*Aedes albopictus*	Ecuador	Cachaco	0,8337	-78,4023	07/11/2018	Female	H2	Ovitrap	This study
OK266909	ECU27426	*Aedes albopictus*	Ecuador	Cachaco	0,8337	-78,4023	28/01/2021	Male	H2	Ovitrap	This study
OK266910	ECU27427	*Aedes albopictus*	Ecuador	Cachaco	0,8337	-78,4023	14/01/2021	Male	H2	Ovitrap	This study
OK266911	ECU27428	*Aedes albopictus*	Ecuador	Guadual	0,8918	-78,5022	07/01/2021	Female	H2	Rubber tire	This study
OK266912	ECU27429	*Aedes albopictus*	Ecuador	Guadual	0,8918	-78,5022	07/01/2021	Female	H2	Rubber tire	This study
OK266913	ECU27430	*Aedes albopictus*	Ecuador	Guadual	0,8918	-78,5022	28/01/2021	Male	H2	Ovitrap	This study
OK266914	ECU27431	*Aedes albopictus*	Ecuador	Guadual	0,8918	-78,5022	28/01/2021	Female	H2	Ovitrap	This study
OK266915	ECU27432	*Aedes albopictus*	Ecuador	Guadual	0,8918	-78,5022	28/01/2021	Female	H2	Rubber tire	This study
OK266916	ECU27433	*Aedes albopictus*	Ecuador	Guadual	0,8918	-78,5022	28/01/2021	Female	H2	Ovitrap	This study
OK266917	ECU27434	*Aedes albopictus*	Ecuador	Guadual	0,8918	-78,5022	28/01/2021	Female	H2	Rubber tire	This study
OK266918	ECU27435	*Aedes albopictus*	Ecuador	Guadual	0,8918	-78,5022	28/01/2021	Male	H2	Ovitrap	This study
OK266919	ECU27436	*Aedes albopictus*	Ecuador	Guadual	0,8918	-78,5022	04/02/2021	Male	H2	Rubber tire	This study
OK266920	ECU27437	*Aedes albopictus*	Ecuador	Guadual	0,8918	-78,5022	28/01/2021	Male	H2	Ovitrap	This study
OK266921	ECU27438	*Aedes albopictus*	Ecuador	Lita	0,8337	-78,4017	20/02/2019	Female	H2	Ovitrap	This study
OK266922	ECU27439	*Aedes albopictus*	Ecuador	Lita	0,8337	-78,4017	20/02/2019	Female	H2	Ovitrap	This study
OK266923	ECU27440	*Aedes albopictus*	Ecuador	Lita	0,8337	-78,4017	20/02/2019	Female	H2	Ovitrap	This study
OK266924	ECU27441	*Aedes albopictus*	Ecuador	Lita	0,8337	-78,4017	20/02/2019	Female	H2	Ovitrap	This study
OK266925	ECU27442	*Aedes albopictus*	Ecuador	Lita	0,8337	-78,4017	20/02/2019	Female	H2	Ovitrap	This study
OK266926	ECU27444	*Aedes albopictus*	Ecuador	Lita	0,8337	-78,4017	20/02/2019	Male	H2	Ovitrap	This study

Table S1. Continue

Accession N°	ECUs	Organism	Country	Locality	Latitude	Longitude	Collection Date	Sex	Haplotype	Trap	Reference
OK266927	ECU27462	*Aedes albopictus*	Ecuador	Lita	0,8337	-78,4017	20/02/2019	Male	H2	Ovitrap	This study
OK266928	ECU27463	*Aedes albopictus*	Ecuador	Lita	0,8337	-78,4017	20/02/2019	Male	H2	Ovitrap	This study
OK266929	ECU27464	*Aedes albopictus*	Ecuador	Lita	0,8337	-78,4017	20/02/2019	Male	H2	Ovitrap	This study
OK266930	ECU27465	*Aedes albopictus*	Ecuador	Lita	0,8337	-78,4017	20/02/2019	Male	H2	Ovitrap	This study
OK266931	ECU27466	*Aedes albopictus*	Ecuador	Lita	0,8337	-78,4017	20/02/2019	Male	H2	Ovitrap	This study
OK266932	ECU27467	*Aedes albopictus*	Ecuador	Lita	0,8337	-78,4017	20/02/2019	Male	H2	Ovitrap	This study
OK266933	ECU27468	*Aedes albopictus*	Ecuador	Guayaquil	-2,1633	-79,8938	20/04/2017	Male	H1	BG Centinet	This study
OK266934	ECU27469	*Aedes albopictus*	Ecuador	Guayaquil	-2,1633	-79,8938	27/04/2017	Male	H1	BG Centinet	This study
OK266935	ECU27470	*Aedes albopictus*	Ecuador	Guayaquil	-2,1633	-79,8938	11/05/2017	Male	H1	Rubber tire	This study
OK266936	ECU27471	*Aedes albopictus*	Ecuador	Guayaquil	-2,1633	-79,8938	28/04/2017	Male	H1	BG Centinet	This study
OK266937	ECU27472	*Aedes albopictus*	Ecuador	Guayaquil	-2,1633	-79,8938	26/04/2017	Female	H1	BG Centinet	This study
OK266938	ECU27473	*Aedes albopictus*	Ecuador	Guayaquil	-2,1633	-79,8938	27/04/2017	Female	H1	BG Centinet	This study
OK266939	ECU27474	*Aedes albopictus*	Ecuador	Guayaquil	-2,1633	-79,8938	09/05/2017	Female	H1	BG Centinet	This study
OK266940	ECU27475	*Aedes albopictus*	Ecuador	Guayaquil	-2,1633	-79,8938	04/05/2017	Female	H1	Hollow rock	This study
OK266941	ECU27476	*Aedes albopictus*	Ecuador	Guayaquil	-2,1633	-79,8938	21/04/2017	Female	H1	BG Centinet	This study
OK266942	ECU27303	*Aedes albopictus*	Ecuador	Guayaquil	-2,1633	-79,8938	09/05/2017	Female	H1	Immature	This study
OK266943	ECU27317	*Aedes albopictus*	Ecuador	Francisco de Orellana	-0,4412	-77,0048	30/05/2018	Female	H1	Metal tube	This study
OK266944	ECU27309	*Aedes albopictus*	Ecuador	Francisco de Orellana	-0,4412	-77,0048	30/05/2018	Female	H1	Metal tube	This study
OK266945	ECU27313	*Aedes albopictus*	Ecuador	Francisco de Orellana	-0,4412	-77,0048	30/05/2018	Male	H1	Metal tube	This study
OK266946	ECU27310	*Aedes albopictus*	Ecuador	Francisco de Orellana	-0,4412	-77,0048	30/05/2018	Male	H1	Metal tube	This study
OK266947	ECU27311	*Aedes albopictus*	Ecuador	Francisco de Orellana	-0,4412	-77,0048	30/05/2018	Male	H1	Metal tube	This study
OK266948	ECU27314	*Aedes albopictus*	Ecuador	Francisco de Orellana	-0,4412	-77,0048	30/05/2018	Male	H1	Metal tube	This study
OK266949	ECU27316	*Aedes albopictus*	Ecuador	Francisco de Orellana	-0,4412	-77,0048	30/05/2018	Male	H1	Metal tube	This study

Table S2. Reported *Aedes albopictus* sequences for COI mitochondrial marker used for phylogenetic relationship analysis

Accession N°	Organism	Locality	Cluster	Haplotype	Reference
KJ012173.1	*Culex quinquefasciatus*	Turkey	Outgroup	---	Gunay et al., unpublished
MK890402.1	*Aedes aegypti*	Ecuador: Guayaquil	Outgroup	---	Ponce et al., 2021
MK890435.1	*Aedes aegypti*	Ecuador: Lita	Outgroup	---	Ponce et al., 2021
KX886324.1	*Aedes albopictus*	China: Hainan	Ancestral	---	Guo and Zheng, unpublished
KF406456.1	*Aedes albopictus*	Pakistan: Punjab	Ancestral	---	Ashfaq et al., 2014
MK505590.1	*Aedes albopictus*	Malaysia	Ancestral	---	Hernandez-Triana et al., 2019
AB690835.1	*Aedes albopictus*	Japan	Base	---	Kuwata et al., 2012
AB907801.1	*Aedes albopictus*	Panama	Base	H72	Futami et al., 2015
OK266937.1	*Aedes albopictus*	Ecuador: Guayaquil	Base	---	This study
HQ398900.1	*Aedes albopictus*	Viet Nam	Base	---	Cook et al., 2010
JQ412504.1	*Aedes albopictus*	Turkey: Edirne, Ipsala	Base	---	Oter et al., 2013
JX679374.1	*Aedes albopictus*	Italy	Base	---	Shaikevich and Talbalaghi, 2013
KC690927.1	*Aedes albopictus*	Singapore	Base	H32	Zhong et al., 2013
KF406409.1	*Aedes albopictus*	Pakistan	Base	---	Ashfaq et al., 2014
KM613097.1	*Aedes albopictus*	Thailand	Base	---	Ruangsittichai et al., unpublished
KU319443.1	*Aedes albopictus*	Spain	Base	H01	Brustolin et al., unplublished
KU522419.1	*Aedes albopictus*	Morocco: Rabat	Base	---	Bennouna et al., unpublished
KU926300.1	*Aedes albopictus*	Mauritius	Base	H01	Walther et al., 2016
KX266678.1	*Aedes albopictus*	China: Shanghai	Base	H42	Gao et al., 2016 (Direct submission)
KX383931.1	*Aedes albopictus*	Albania	Base	A1a2a1	Battaglia et al., 2016
KX886284.1	*Aedes albopictus*	China: Fujian	Base	---	Guo and Zheng, unpublished
KX886311.1	*Aedes albopictus*	China: Hainan	Base	---	Guo and Zheng, unpublished
MF622084.1	*Aedes albopictus*	USA: Ohio	Base	H3	Giordano et al., unpublished
MF990905.1	*Aedes albopictus*	Portugal	Base	---	Osorio et al., 2018
MG871392.1	*Aedes albopictus*	South Korea	Base	H28	Lee, E. et al., 2020
MH817490.1	*Aedes albopictus*	Russia: Adler	Base	---	Fedorova and Shaikevich, unpublished
MK297326.1	*Aedes albopictus*	India	Base	---	Lakshmi et al., unpublished
MK439903.1	*Aedes albopictus*	Czech Republic	Base	---	Kurikova, unpublished
MK505570.1	*Aedes albopictus*	Montenegro: Radovici Village	Base	---	Hernandez-Triana et al., 2019

Table S2. Continue

Accession N°	Organism	Locality	Cluster	Haplotype	Reference
MK518354.1	*Aedes albopictus*	Serbia: Novi Sad	Base	---	Gojkovic et al., 2019
MK575475.1	*Aedes albopictus*	Brazil	Base	---	Lorenz et al., unpublished
MN005054.1	*Aedes albopictus*	Greece	Base	---	Bisia et al., unpublished
MN080720.1	*Aedes albopictus*	Lao PDR	Base	---	Motoki et al., 2019
MN103394.1	*Aedes albopictus*	Austria: Tyrol, Kufstein	Base	---	Fuehrer and Walder, unpublished
MN509201.1	*Aedes albopictus*	USA: New York	Base	---	Lee, A. et al., 2019 (Direct submission)
MN997607.1	*Aedes albopictus*	Colombia: Choco, Bellavista	Base	---	Acosta et al., 2020 (Direct submission)
OK266946	*Aedes albopictus*	Ecuador: Francisco de Orellana	Base	---	This study
AB907796.1	*Aedes albopictus*	Costa Rica	1	---	Futami et al., 2015
KM457524.1	*Aedes albopictus*	Netherlands	1	---	van de Vossenberg et al., 2015
KX886334.1	*Aedes albopictus*	China: Hainan	2	---	Guo and Zheng, unpublished
MN080732.1	*Aedes albopictus*	Lao PDR	2	H67	Motoki et al., 2019
LC054326.1	*Aedes albopictus*	Japan	3	---	Maekawa et al., 2016
MG871396.1	*Aedes albopictus*	South Korea	3	---	Lee, E. et al., 2020
KC690954.1	*Aedes albopictus*	USA: Texas	4	---	Zhong et al., 2013
MN103393.1	*Aedes albopictus*	Austria: Tyrol, Weer Sud	4	---	Fuehrer and Walder, unpublished
KM457544.1	*Aedes albopictus*	Netherlands	5	---	van de Vossenberg et al., 2015
KU319445.1	*Aedes albopictus*	Spain	5	---	Brustolin et al., unplublished
KF406404.1	*Aedes albopictus*	Pakistan: Punjab	6	---	Ashfaq et al., 2014
MG572237.1	*Aedes albopictus*	Bangladesh	6	---	Aslam et al., unpublished
KU495082.1	*Aedes albopictus*	Australia	7	---	Batovska et al., 2016
MH025948.1	*Aedes albopictus*	Republic of the Congo: Gamboma	7	---	Kamgnag et al., unpublished
MH330192.1	*Aedes albopictus*	Sri Lanka: Negombo	7	---	Hapuarachchi et al., unpublished
KX886314.1	*Aedes albopictus*	China: Hainan	8	---	Guo and Zheng, unpublished
KX886327.1	*Aedes albopictus*	China: Hainan	8	---	Guo and Zheng, unpublished
MH330189.1	*Aedes albopictus*	Sri Lanka: Kiribathgoda	8	---	Hapuarachchi et al., unpublished
OK266909.1	*Aedes albopictus*	Ecuador: Cachaco	9	---	This study
OK266918.1	*Aedes albopictus*	Ecuador: Guadual	9	---	This study
KC690922.1	*Aedes albopictus*	Singapore	9	H27	Zhong et al., 2013

Table S2. Continue

Accession N°	Organism	Locality	Cluster	Haplotype	Reference
OK266923.1	*Aedes albopictus*	Ecuador: Lita	9	---	This study
MF148286.1	*Aedes albopictus*	Malaysia: Selangor	9	H38	Adilah-Amrannudin et al., unpublished
MH921569.1	*Aedes albopictus*	Cameroon	9	H02	Kamgang et al., unpublished
MN997608.1	*Aedes albopictus*	Colombia: Antioquia, Murind	9	---	Acosta et al., 2020 (Direct submission)
MN997609.1	*Aedes albopictus*	Colombia: Antioquia, Vigia del Fuerte	9	---	Acosta et al., 2020 (Direct submission)
MN997610.1	*Aedes albopictus*	Colombia: Choco, Bellavista	9	---	Acosta et al., 2020 (Direct submission)
MN997611.1	*Aedes albopictus*	Colombia: Antioquia, Murindo	9	---	Acosta et al., 2020 (Direct submission)
MT345357.1	*Aedes albopictus*	Democratic Republic of the Congo: Kinshasa	9	---	Wat'senga Tezzo et al., 2021
MT345358.1	*Aedes albopictus*	Democratic Republic of the Congo: Kinshasa	9	---	Wat'senga Tezzo et al., 2021

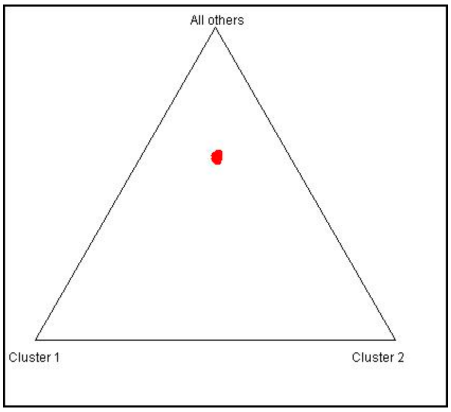

Figure S1. Triangular plot produced by the Structure, showing only one genetic cluster in the Ecuadorian populations of *Aedes albopictus*.

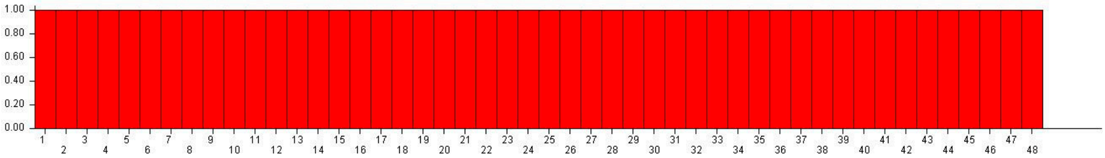

Figure S2. Bar plot produced by the Structure, showing only one genetic cluster in the Ecuadorian populations of *Aedes albopictus*.
